# α-Melanocyte-Stimulating Hormone Protects Retinal Vascular Endothelial Cells from Oxidative Stress and Apoptosis in a Rat Model of Diabetes

**DOI:** 10.1371/journal.pone.0093433

**Published:** 2014-04-02

**Authors:** Lijuan Zhang, Lijie Dong, Xun Liu, Yuanfeng Jiang, Lingjun Zhang, Xiaomin Zhang, Xiaorong Li, Yan Zhang

**Affiliations:** Tianjin Medical University Eye Hospital/Eye Institute, Tianjin, China; University of Florida, United States of America

## Abstract

**Aims:**

Oxidative stress and apoptosis are among the earliest lesions of diabetic retinopathy. This study sought to examine the anti-oxidative and anti-apoptotic effects of α-melanocyte-stimulating hormone (α-MSH) in early diabetic retinas and to explore the underlying mechanisms in retinal vascular endothelial cells.

**Methods:**

Sprague-Dawley rats were injected intravenously with streptozocin to induce diabetes. The diabetic rats were injected intravitreally with α-MSH or saline. At week 5 after diabetes, the retinas were analyzed for reactive oxygen species (ROS) and gene expression. One week later, the retinas were processed for terminal deoxynucleotidyl transferase dUTP nick-end labeling staining and transmission electron microscopy. Retinal vascular endothelial cells were stimulated by high glucose (HG) with or without α-MSH. The expression of *Forkhead box O* genes (*Foxos*) was examined through real-time PCR. The *Foxo4* gene was overexpressed in endothelial cells by transient transfection prior to α-MSH or HG treatment, and oxidative stress and apoptosis were analyzed through CM-H2DCFDA and annexin-V assays, respectively.

**Results:**

In diabetic retinas, the levels of H_2_O_2_ and ROS and the total anti-oxidant capacity were normalized, the apoptotic cell number was reduced, and the ultrastructural injuries were ameliorated by α-MSH. Treatment with α-MSH also corrected the aberrant changes in *eNOS*, *iNOS*, *ICAM-1*, and *TNF-α* expression levels in diabetic retinas. Furthermore, α-MSH inhibited *Foxo4* up-regulation in diabetic retinas and in endothelial cells exposed to HG, whereas *Foxo4* overexpression abrogated the anti-oxidative and anti-apoptotic effects of α-MSH in HG-stimulated retinal vascular endothelial cells.

**Conclusions:**

α-MSH normalized oxidative stress, reduced apoptosis and ultrastructural injuries, and corrected gene expression levels in early diabetic retinas. The protective effects of α-MSH in retinal vascular endothelial cells may be mediated through the inhibition of *Foxo4* up-regulation induced by HG. This study suggests an α-MSH-mediated potential intervention approach to early diabetic retinopathy and a novel regulatory mechanism involving *Foxo4*.

## Introduction

Diabetic retinopathy (DR) is the vascular and neural injury of the retina caused by metabolic disorders in diabetes. DR has no or mild symptoms at early stages; however, if not properly treated, DR may progress to the advanced stage, during which severe pathologies often lead to irreversible blindness [1]. Moreover, the current therapeutic modalities, including laser photocoagulation [2] and vitrectomy [3], are used to relieve symptoms, and the therapeutic effects are not satisfactory. Therefore, a novel and effective intervention approach at the early stages of DR that targets the initial injury and direct cause of the disease is needed.

Research in diabetic patients and animal models demonstrates that the earliest injuries in the retina occur in microvessel endothelial cells and manifest as the breakdown of the retinal blood barrier and leukostasis [4], [5]. If the metabolic disorder is not corrected, apoptosis of microvessel endothelial cells and loss of pericytes may occur [6], after which the typical pathological changes in non-proliferative diabetic retinopathy are observed [7], [8]. It appears clear that high glucose (HG) levels associated with metabolic disorders in diabetes are the direct cause of endothelial cell injury in retinal microvessels. However, reports in the literature indicate that if type I diabetic patients are not subjected to appropriate glycemic control within the first 5 years of the disease (HbA1c >7%), even though the glycemic level later returns to normal, the endothelial function cannot be restored [9]. Similarly, if blood glucose levels are not controlled (HbA1c >11%) in streptozocin (STZ)-induced type I diabetic rats during the initial 6 months of the disease, the apoptotic endothelia in retinal vessels and acellular capillaries continue to increase, even 7 months after the glycemic level normalizes [10]. This phenomenon, known as “metabolic memory”, suggests that HG is not the direct cause of retinal endothelial cell injury in diabetes [11]. On the other hand, the high levels of glucose within retinal vascular endothelial cells stimulate the mitochondrial electron transport chain to produce a large amount of reactive oxygen species (ROS), which, in turn, generate an intracellular milieu of oxidative stress [12]. It is generally believed that oxidative stress directly activates a cascade of biochemical and molecular events, including polyol flow and the hexosamine pathway, advanced glycation end products and their receptor, proinflammatory factors, and protein kinase C signaling, which lead to retinal vascular endothelial cell injury [11]. Therefore, an effective intervention approach for early DR would target HG-induced oxidative stress in retinal vascular endothelial cells.

α-melanocyte-stimulating hormone (α-MSH) is a widely distributed 13-amino acid peptide that is derived from the proteolytic cleavage of proopiomelanocortin [13]. After posttranslational modifications at both termini, α-MSH binds to 5 subtypes of G protein-coupled receptors (melanocortin receptors, MC1R-MC5R) [14]. α-MSH and its receptor system activate the cAMP-PKA and Ras-raf-MEK-MAPK signaling pathways to mediate biological functions [15]. MC3R and MC4R regulate physiological metabolism in humans [16] and animals [17]. α-MSH is also present in the serum of type I diabetic patients, although its concentration is not significantly elevated due to greater individual variability compared with healthy controls [18]. In the eye, α-MSH modulates the immune response and maintains the immunoprivilege under physiological conditions [19]; it also exerts therapeutic effects in diseased models. For example, α-MSH suppresses inflammation and maintains an intact retina in infectious [20] and autoimmune [21] uveitis, respectively. Intravitreal injection of an α-MSH analog protects photoreceptor cells from death in a rat model of retinal dystrophy in a dose-dependent manner [22]. It is also notable that α-MSH suppresses the oxidative stress induced by ultraviolet radiation in skin keratinocytes and melanocytes via the MC1R-mediated cAMP-PKA pathway, independent of melanin genesis [23]–[25]. Therefore, it was of interest to study, for the first time, whether α-MSH, applied intravitreally, exerts anti-oxidative and cytoprotective effects in a rat model of early DR.

The forkhead box proteins of O class (FoxOs) proteins are a family of transcription factors that control a variety of physiological processes, including cell proliferation, differentiation, DNA damage, apoptosis [26], [27], and the defense system under oxidative stress [28]. Four isoforms of the FoxOs, including FoxO1, FoxO3, FoxO4, and FoxO6, have been identified in mammals and have overlapping functions. A recent study showed that the endothelial cell-specific ablation of *Foxo1*, *3* and *4* genes protected low-density lipoprotein receptor-knockout mice from atherosclerosis under diabetic-like conditions [29], indicating that FoxO4 is at least one of the effectors causing endothelial dysfunction and damage. Furthermore, in the cultured podocytes of glomerular capillaries that share structural similarities to retinal microvessels, FoxO4 is the only member of the FoxOs that is activated by advanced glycation end products and mediates apoptosis of these cells [30]. Thus, FoxO4 could be the main pathogenic factor mediating endothelial damage under hyperglycemia. However, FoxO4 expression and activity are tightly regulated at multiple levels and in distinctive cellular compartments [31]. For instance, FoxO4 is phosphorylated by activation of the PI3K/Akt pathway, the nuclear translocation of the phosphorylated transcription factor is then inhibited, and the transcription of its downstream pro-apoptotic and proinflammatory genes cannot be activated [32]. Furthermore, the binding of α-MSH to the major melanocortin receptors, including MC3R [33], MC4R [34], and MC5R [35], can elicit PI3K activation. Hence, we hypothesized that α-MSH may exert anti-oxidative and anti-apoptotic effects in retinal vascular endothelial cells through inhibiting the transcription factor FoxO4. Moreover, post-translational regulation of FoxO4, such as acetylation, ubiquitination, and translocation, has been extensively studied [36], the regulation of *FoxO4* at the transcriptional level is less clear. Therefore, in this study, we first investigated the protective effects of α-MSH in the retina of early diabetic rats and then examined the transcriptional regulation of *FoxO4* in both diabetic retinas and cell cultures. Finally, we tested the hypothesis in HG-stimulated retinal vascular endothelial cells, a cell model that recapitulates the STZ-induced diabetic condition. Our results demonstrated that intravitreal injections of α-MSH exerted anti-oxidative and anti-apoptotic effects in early diabetic retinas; the transcript levels of *FoxO4* were up-regulated under diabetic conditions, and this up-regulation was inhibited by α-MSH. The results also suggest that the protective effects of α-MSH in the retinal vascular endothelial cells may be due to its inhibition of the *FoxO4* up-regulation induced by HG.

## Materials and Methods

### Ethics statement

This study was performed in accordance with the Guide for the Care and Use of Laboratory Animals of the National Institutes of Health. The protocol was approved by the Institutional Animal Care and Use Committee (IACUC) of Tianjin Medical University (Permit Number: SYXK 2009-0001). All surgeries were performed under chloral hydrate anesthesia, and all efforts were made to minimize suffering.

### Diabetic induction and intravitreal injections of α-MSH

Male Sprague-Dawley (SD) rats (body weight, 200–250 g) at 5–6 weeks of age were purchased from the animal facility at the Chinese Academy of Medical Science of Radiation (Tianjin, China). The animals had free access to food and water and were maintained under a 12∶12 h light-dark cycle at 22–25°C with relative humidity of 40∼70%. Diabetes was induced by a tail vein injection of 2% streptozocin (STZ; dissolved in sodium citrate buffer, pH 4.5; Amresco Chemical Co., Solon, OH, USA) at a dose of 45 mg/kg. Blood glucose levels were monitored 72 h later; only animals with a blood glucose level >20 mM were included in the diabetic mellitus (DM) group for the following experiments. The normal control group (normal) was injected with sodium citrate buffer. The body weight and blood glucose levels were monitored immediately before diabetic induction and on day 3 and weeks 1, 2, 3 and 4 after induction.

The intravitreal injection of α-MSH was performed at weeks 1 and 3 after diabetic induction. Briefly, α-MSH (10 μg/3 μl, catalog number 05-23-0751, Merck, Whitehouse Station, NJ, USA) was injected with a 30-gauge needle mounted on a Hamilton syringe (Sigma, Shanghai, China) at 2 mm posterior to limbus in the right eye of diabetic animals; these animals were designated as the α-MSH+DM group. The same volume of sterilized normal saline (0.9% NaCl) was injected intravitreally into the left eyes of the diabetic and non-diabetic rats constituting the diabetic non-treatment (DM) and normal control (normal) groups, respectively.

### H_2_O_2_ and ROS levels and total antioxidant capacity in the retina

At week 5 after diabetic induction, retina samples were collected, snap frozen in liquid nitrogen, and stored at −80°C for protein and RNA analyses. Total protein was extracted with a Tissue Protein Extraction Kit (CWBIO, Beijing, China), and the protein concentration was determined by a Bicinchoninic Acid (BCA) Protein Assay Kit (CWBIO, Beijing, China). Twenty-five microliters of the 5-fold diluted protein samples and the serially diluted albumin standards were incubated with BCA reagent at 37°C for 30 min, and absorbance at 562 nm was measured. The protein concentration was calculated from a standard curve. The H_2_O_2_ content was measured using a Hydrogen Peroxide Assay Kit (Beyotime Institute of Biotechnology, Shanghai, China) according to the company's protocol. Briefly, 50 μl protein samples were incubated with the reagent at room temperature for 30 min, and the absorbance at 560 nm was measured. The H_2_O_2_ concentration (mM) was calculated from a standard curve. The ROS levels were examined by a ROS Assay Kit (Nanjing Jiancheng Bioengineering Institute, Nanjing, China) following the manufacturer's instructions. One hundred and ninety microliter protein samples were incubated with 5 μl freshly prepared solution containing 1 mM 2′,7′-dichlorofluorescin diacetate. The fluorescence intensity was measured with the excitation wavelength at 500 nm and the emission wavelength at 530 nm. The total antioxidant capacity was measured using a Total Antioxidant Capacity Assay Kit (Beyotime Institute of Biotechnology, Shanghai, China) based on the ferric reducing ability of plasma (FRAP) method. Five microliter protein samples and the serially diluted ferrous sulfate (FeSO_4_) solutions (serving as standards) were incubated with 180 μl FRAP solution at 37°C for 5 min. The absorbance at 593 nm was measured. The total antioxidant capacity equivalent to certain concentration (mM) of the FeSO_4_ standard solution was calculated from a standard curve. All absorbance and fluorescence intensities were measured by an Infinite 200 PRO Multimode Microplate Reader (Tecan Group Ltd., Männedorf, Switzerland). The calculated levels of H_2_O_2_ and ROS and the total antioxidant capacity were normalized to the protein concentration in each retina sample.

### Trypsin-digested retinal vessel preparation

At week 6 following diabetic induction, the rats were euthanized by an excessive dose of 10% chloral hydrate. The eyes were removed and fixed in 4% paraformaldehyde solution (PFA; Sigma, Shanghai, China) for 72 h, then transferred to 0.01 M phosphate-buffered saline (PBS; pH 7.4) for 24 h. The retinas were carefully dissected and subjected to trypsin digestion, as previously described [37] with minor modifications. Briefly, following digestion with 3% crude trypsin (BD, Franklin Lakes, NJ, USA, in 0.1 M Tris and 0.2 M NaF solution, pH 7.8), the retinas were transferred to distilled water, and the adherent tissues were separated from the vessels by flushing with water drop-wise using a 1-ml syringe with a curved needle. The transparent retinal vascular network was then mounted on a glass slide using a self-made, blunt-tip, glass rod. The slides were air dried and stored at 4°C.

### Perfusion and retinal cryosections

At week 6 after diabetic induction, the rats were deeply anesthetized with 10% chloral hydrate and thoroughly perfused via the left ventricle with ice-cold PBS and then with 1% PFA (100 ml/kg). The eyes were postfixed and dehydrated in graded sucrose solutions. The anterior segments were removed. The eye cups were embedded in Tissue-Tek O.C.T. compound (Sakura Finetek, Torrance, CA, USA), frozen in dry ice, and sagittally sectioned on a cryostat. The sections (8 μm) were mounted on glass slides and stored at −20°C.

### Terminal deoxynucleotidyl transferase dUTP nick-end labeling of retinal cryosections and vasculatures

Terminal deoxynucleotidyl transferase dUTP nick-end labeling (TUNEL) staining was performed on retinal cryosections and on vasculature preparations using an In Situ Cell Death Detection Kit, Fluorescein (Roche Diagnostics; Branford, CT, USA). The reaction mixture containing DNase I or lacking terminal deoxynucleotidyl transferase was used to generate the positive and negative control, respectively. Following TUNEL staining, a cover slip with the ProLong Gold Antifade reagent (with DAPI, Life Technologies, Beijing, China) was mounted on each slide. The stained sections were observed under a fluorescence microscope (BX51; Olympus Optical Co. Ltd., Tokyo, Japan). The images were captured by the cellSens Standard electronic system (Olympus Optical Co. Ltd., Tokyo, Japan). Ten sections that represent comparable positions from the peripheral to the central retina were chosen for each retina. Images at appropriate magnifications were captured for each retinal section with identical optical parameters. The images at lower magnifications were used to quantify the estimated representation of the number of TUNEL-positive cells per retinal section. The fluorescent signals in the nuclei with intensities stronger than the non-specific background were considered positive. The representative images were selected from the higher magnification images for better visualization.

### Transmission electron microscopy

At week 6 after diabetic induction, the eyes were processed for transmission electron microscopy, as previously described [38]. Briefly, the eye cups were fixed with pre-chilled 2.5% glutaraldehyde solution (pH 7.4) and 1% osmium tetroxide solution and then dehydrated in ethanol. The samples were embedded in 812 resin (Electron Microscopy Sciences, Hatfield, PA, USA) after treating with epoxy propane. Ultra-thin sections (±50 nm) were stained with uranyl acetate and lead citrate and observed under a Hitachi-7500 transmission electron microscope (Hitachi, Tokyo, Japan). The images were captured by the MegaView digital electron microscopy system (Olympus Soft Imaging Solutions Corp., Lakewood, CO, USA).

### RT-PCR analyses

Total RNA was extracted from the retina samples using a GeneJET RNA Purification Kit (Thermo Fisher Scientific, Beijing, China). The concentration and purity of the RNA were determined by a Nanodrop 2000 (Thermo Fisher Scientific, Waltham, MA, USA). Total RNA (250 ng) was digested with DNase I, reverse transcribed in a 20 μl reaction mixture using a RevertAid cDNA Synthesis Kit (Thermo Fisher Scientific, Beijing, China).

Regular PCR was conducted in a GeneAmp PCR System 2400 (PerkinElmer, Waltham, MA, USA) using GoTaq Green 2X Master Mix (Thermo Fisher Scientific, Beijing, China), the cDNA template from diabetic rat retina, and specific primers for the *MCR* genes (Table 1). PCR assays using rat liver or hypothalamus cDNA and water as templates were included as the positive and negative controls, respectively. The regular PCR reaction began with a 5-min denaturation at 94°C followed by 35 cycles of denaturation at 94°C for 30 s, annealing at 60°C for 30 s, and extension at 72°C for 1 min, with a final extension at 72°C for 7 min. The PCR products were visualized via gel electrophoresis.

**Table 1:** PCR Primers used in this study.

Gene	Species	NCBI accession #	PCR purpose	Primer sequence
*eNOS*	rat	NM_021838.2	Real-time	F: 5′-TCTGCGGCGATGTCACTATG-3′
				R: 5′-CATGCCGCCCTCTGTTG-3′
*iNOS*	rat	NM_012611.3	Real-time	F: 5′-TGCTAATGCGGAAGGTCATG-3′
				R: 5′-GCTTCCGACTTTCCTGTCTCA-3′
*nNOS*	rat	NM_052799.1	Real-time	F: 5′-CCAATGTTCACAAAAAACGAGTCT-3′
				R: 5′-TCGGCTGGACTTAGGGCTTT-3′
*ICAM-1*	rat	NM_012967.1	Real-time	F: 5′-CGGGAGATGAATGGTACCTACAA-3′
				R: 5′-TGCACGTCCCTGGTGATACTC-3′
*TNF-α*	rat	NM_012675.3	Real-time	F: 5′-ACAAGGCTGCCCCGACTAC-3′
				R: 5′- CTCCTGGTATGAAATGGCAAATC-3′
*GAPDH*	rat	NM_017008.4	Real-time	F: 5′-TGTGTCCGTCGTGGATCTGA-3′
				R: 5′-CCTGCTTCACCACCTTCTTGA-3′
*MC1R*	rat	AB626606.1	Detection	F: 5′-GAGGCTTCTGGGTTCTCTCA-3′
				R: 5′- CAGCACCTCCTTGAGTGTCA-3′
*MC3R*	rat	NM_001025270.3	Detection	F: 5′-CTTATCCGACGCTGCCTAAC-3′
				R: 5′-ACCGCAGAGAATCTCCTTGA-3′
*MC4R*	rat	NM_013099.2	Detection	F: 5′-CTTCCCTCCACCTCTGGAAC-3′
				R: 5′-CCCAGGGGGTAGAAACAGAT-3′
*MC5R*	rat	NM_013182.2	Detection	F: 5′-CCTCGGAGGACAACATCCTA-3′
				R: 5′-GCCAAGGAGCGTACAAGTTC-3′
*Foxo1*	rat	NM_001191846.2	Real-time	F:5′-CATGCACAGCAAACTTCTTCAGT-3′
				R: 5′-AGATGTGTGAGGCATGGTGTTC-3′
*Foxo3*	rat	NM_001106395.1	Real-time	F: 5′-CTCCATCCGCCACAACCT-3′
				R: 5′-GCCAGTCCCTTCGTTCTGAA-3′
*Foxo4*	rat	NM_001106943.1	Real-time	F: 5′-CTCGCCCAGATATACGAATGG-3′
				R: 5′-GCCGAGCTGTTGCTGTCA-3′
*Foxo6*	rat	XM_001057233.4	Real-time	F: 5′-ACTCCATCCGGCACAACCT-3′
				R: 5′-TTGCCAGTCCCCTCATTCTG-3′
*Foxo1*	monkey	XM_001088437.2	Real-time	F: 5′-GCATCCATGGACAACAATAGTAAATT-3′
				R: 5′-GCCAGACTGGAGGGATGCT-3′
*Foxo3*	monkey	XM_001093593	Real-time	F: 5′-ACCCGCGCCATCGAA-3′
				R: 5′-GCACCATCCACTCGTAGATCTG-3′
*Foxo4*	monkey	XM_003917852.1	Real-time	F: 5′-TCAGCCAGGCCATTGAAAG-3′
				R: 5′-CGGACCATCCACTCGTAGATC-3′
			Expression	5′-ATGGATCCGGGGAATGAGAAT-3′
				5′-TCAGGGATCTGGCTCAAAGTT-3′
*Foxo6*	monkey	XM_003891680.1	Real-time	F: 5′-CCGGCTGGAAGAACTCCAT-3′
				R: 5′-TCGTTCTGCACACGGATGA-3′

Real-time PCR was performed using SYBR Green FastStart 2X Master Mix (Roche, Beijing, China), cDNA template, and gene specific primers (Table 1) in a HT7900 Real-Time PCR System (Applied Biosystems, Foster City, CA, USA). The target rat genes *endothelial nitric oxide synthase* (*eNOS*), *inducible nitric oxide synthase* (*iNOS*), *neuronal nitric oxide synthase* (*nNOS*), *intercellular adhesion molecule-1* (*ICAM-1*), and *tumor necrosis factor-α* (*TNF-α*), as well as rat *Foxo1*, *3*, *4*, and *6*, were amplified. The *glyceraldehyde 3-phosphate dehydrogenase* (*GAPDH*) gene is expressed at a constant level in most cells and has been used as a reference for gene expression analyses in the retina of STZ-induced diabetic rats [39], [40]; therefore, the *GAPDH* gene was selected as the reference in this study. The real-time PCR program consisted of a 2-min incubation at 50°C and a 10-min denaturation at 95°C, followed by 40 cycles of 15 s of denaturation at 95°C and 1 min of annealing/extension at 60°C. The dissociation stage was added to confirm amplicon specificity. The cDNA content of the target genes in each retina was normalized to that of *GAPDH* gene. The relative expression levels of the target genes were analyzed using the comparative threshold cycle (2^−ΔΔCt^) method.

### Cell viability and gene expression analyses of the retinal vascular endothelial cells stimulated with HG

A monkey retinal vascular endothelial cell line RF/6A (Chinese Academy of Science, Shanghai, China) was maintained in a complete culture medium consisting of RPMI 1640 (glucose at 11.1 mM; Life Technologies, Beijing, China), 10% fetal bovine serum (Life Technologies, Beijing, China), 100 U/ml penicillin, 100 μg/ml streptomycin (Life Technologies, Beijing, China) and 2 mM L-glutamine (Life Technologies, Beijing, China) at 37°C and 5% CO_2_ in a humidified incubator. Cells were seeded at a density of 2×10^5^/well in 24-well plates and stimulated the following day with RMPI 1640 media containing 25 mM HG.

To test cell viability, α-MSH at concentrations of 1 and 10 nM and 0.1, 1, and 10 μM were incubated with the cells 30 min before and during the HG stimulation, and the cells cultured with plain RPMI 1640 (11.1 mM glucose) were included as normal controls. Eight hours after HG stimulation, cell viability was measured using a Cell Counting Kit-8 (Dojindo Laboratories, Kumamoto, Japan). The cells were incubated with plain RPMI 1640 containing 10% CCK-8 solution for 1 h at 37°C; the absorbance at 450 nm was measured using an Infinite 200 PRO Multimode Microplate Reader (Tecan Group Ltd., Männedorf, Switzerland). The absorbance of the cells under treatment was expressed as the percentage (%) of the normal controls.

For the gene expression analyses, RF/6A cells were seeded and stimulated with HG as described above. α-MSH, according to the results of cell viability test, was used at 0.1 μM to treat the cells 30 min prior to and during HG stimulation. The cells incubated with plain RMPI 1640 served as the normal controls. Eight hours after HG stimulation, the cells were harvested for real-time RT-PCR using the primers in Table 1 for analyzing the expression of monkey *Foxo1, 3, 4, and 6*.

### Oxidative stress assay of the retinal vascular endothelial cells stimulated with HG

The monkey *Foxo4* coding sequence was amplified from the cDNA of RF/6A cells stimulated with HG (cloning primers listed in Table 1), confirmed by sequencing, and cloned into the expression vector pcDNA3.0. The recombinant and empty vectors were designated as *pc-Foxo4* and *pc*, respectively. The RF/6A cells were transfected with *pc-Foxo4* or *pc* at 90–95% confluency in 24-well plates using X-tremeGENE HP DNA Transfection Reagent (Roche; Beijing, China). The media were changed to complete culture media at 5 h post transfection. Twenty-four hours after transfection, the cells were treated with α-MSH and then HG as described above, or incubated with plain RMPI 1640. Nontransfected cells subjected to α-MSH and/or HG treatment, as well as normal control cells, were included. The culture media were removed 8 h later. The cells were washed twice with pre-warmed PBS and incubated with freshly prepared 5 μM 5-(and-6)-chloromethyl-2′,7′-dichlorodihydrofluorescein diacetate, acetyl ester (CM-H2DCFDA; Life Technologies, Beijing, China) for 30 min at 37°C in the dark. The cells were washed twice again and incubated with PBS for another 15 min at 37°C in the dark. Then, the intracellular fluorescence intensity was measured by an Infinite 200 PRO Multimode Microplate Reader (Tecan Group Ltd., Männedorf, Switzerland) with excitation at 495 nm and emission at 525 nm. The fluorescent images were captured with fixed optical parameters immediately after the intensity measurement using the cellSens Standard electronic system (Olympus Optical Co. Ltd., Tokyo, Japan). For the cells in each culture well, two images were captured in each quadrant of the culture well. The images within each treatment group were compared, and the one approximating the average fluorescence intensity and cell density was selected as the representative for that group. Finally, the total protein of the cells was extracted, and the protein concentration was measured as described above. The intracellular fluorescence intensity was normalized to the total protein concentration (mg/ml) to correct for the potential difference in cell density.

### Apoptosis assay of the retinal vascular endothelial cells stimulated with HG

The RF/6A cells transfected with *pc-Foxo4* or *pc* or without transfection were treated with α-MSH and/or HG or cultured in plain RPMI 1640, as described above. Eight hours after the treatments, the cells were stained with Alexa 488-conjugated annexin V (Alexa Fluor 488 Annexin V Apoptosis Kit; Life Technologies, Beijing, China) according to the company's protocol. The cells were fixed with 1% PFA (Sigma, Shanghai, China), and the percentage of the cells positive for Alexa 488-annexin V staining was analyzed by a FACSCalibur (BD Biosciences, San Jose, CA, USA) equipped with BD CellQuest Pro software (BD Biosciences, San Jose, CA, USA). The gate was set according to the characteristic properties of the RF/6A cells in forward and side scatter. The forward and side scatters were set in a linear scale. Twenty-thousand gated events were acquired for analysis. The RF/6A cells cultured under the complete culture media and not stained with annexin V served as a negative control and were used to set the marker M1. The cell population inside M1 was considered positive for Alexa 488-annexin V staining.

### Statistical analyses

All data were expressed as the mean±SE. Statistical analyses were performed using Statistical Program for Social Sciences 13.0 (SPSS Inc., San Diego, CA, USA). The data were examined using the D'Agostino and Pearson omnibus normality test. The data with a Gaussian distribution were analyzed by one-way ANOVA followed by a *Tukey post-test*, and those with nonparametric distribution by the Kruskal–Wallis test followed by *Dunn*'*s post-test*. A p value less than 0.05 was considered significant.

## Results

### STZ-injected rats exhibited the diabetic phenotype and *MCR* expression in the retina

**Figure 1:**
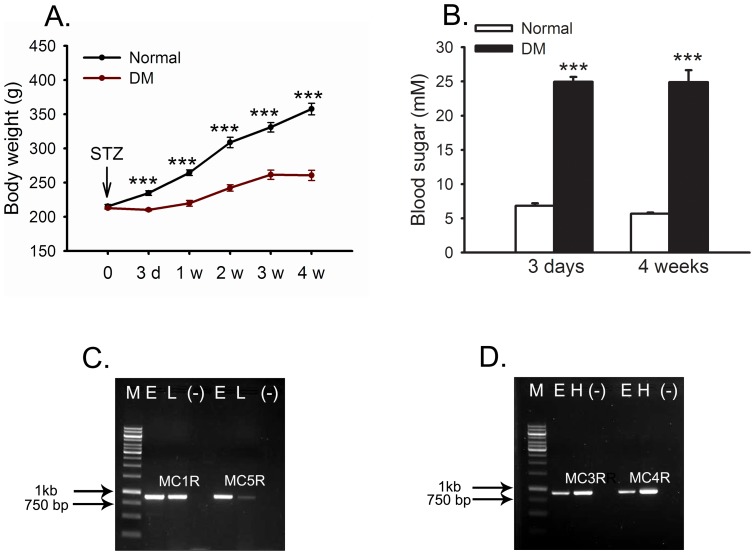
Body weight, blood glucose and MCR expression in STZ-induced diabetic rats. The body weight of the diabetic rats was significantly lower than that of the normal counterparts (A). The blood glucose levels of the diabetic rats averaged at 25 mM at both 3 d and 4 w after diabetic induction and were significantly higher than the normal rats (B). The expression of MC1R and MC5R (C), MC3R and MC4R (D) were detected with the expected size by regular RT-PCR. M: 1 kb DNA ladder, two arrows designate the position of 750 bp and 1 kb fragment in the ladder. E: retina from the eye; L: liver; H: hypothalamus; (-): negative control. *** p<0.001.

The body weight of the DM rats was significantly lower than that of the normal controls (*p*<0.001) at each time point after diabetic induction. The body weight of the diabetic rats also exhibited a slower rate of increase than the normal counterparts, which showed approximately a linear increase along with time (Fig. 1A). However, the body weight in the DM group exhibited no reducing trend during the experiment (Fig. 1A). The blood glucose levels of the rats in the DM group were abnormally high at day 3 and week 4 (24.94±2.79 and 24.88±6.30 mM, respectively) after diabetic induction. The blood glucose levels in the normal control group were significantly lower than the DM group at both time points (*p*<0.001) and were within the normal range (6.82±1.10 mM at 3 d; 5.66±0.40 mM at 4 w; Fig. 1B).

*MC1R*-*MC5R* expression in the diabetic rat retina was detected through RT-PCR. Using retinal cDNA as the template and the receptor-specific primers (Table 1), *MC1R*, *MC3R*, *MC4R*, and *MC5R* gene products were all amplified at the expected size (Fig. 1C, D). The PCR products using liver and hypothalamus cDNA as the template served as positive controls. No PCR product was observed in the negative control using water as the template (Fig. 1C, D). These results suggest that 4 subtypes of melanocortin receptors are expressed in the diabetic rat retina, and α-MSH delivered intravitreally may act upon its cognate receptors to fulfill biological functions.

### α-MSH normalized ROS levels and the total antioxidant capacity in early diabetic retinas

**Figure 2:**
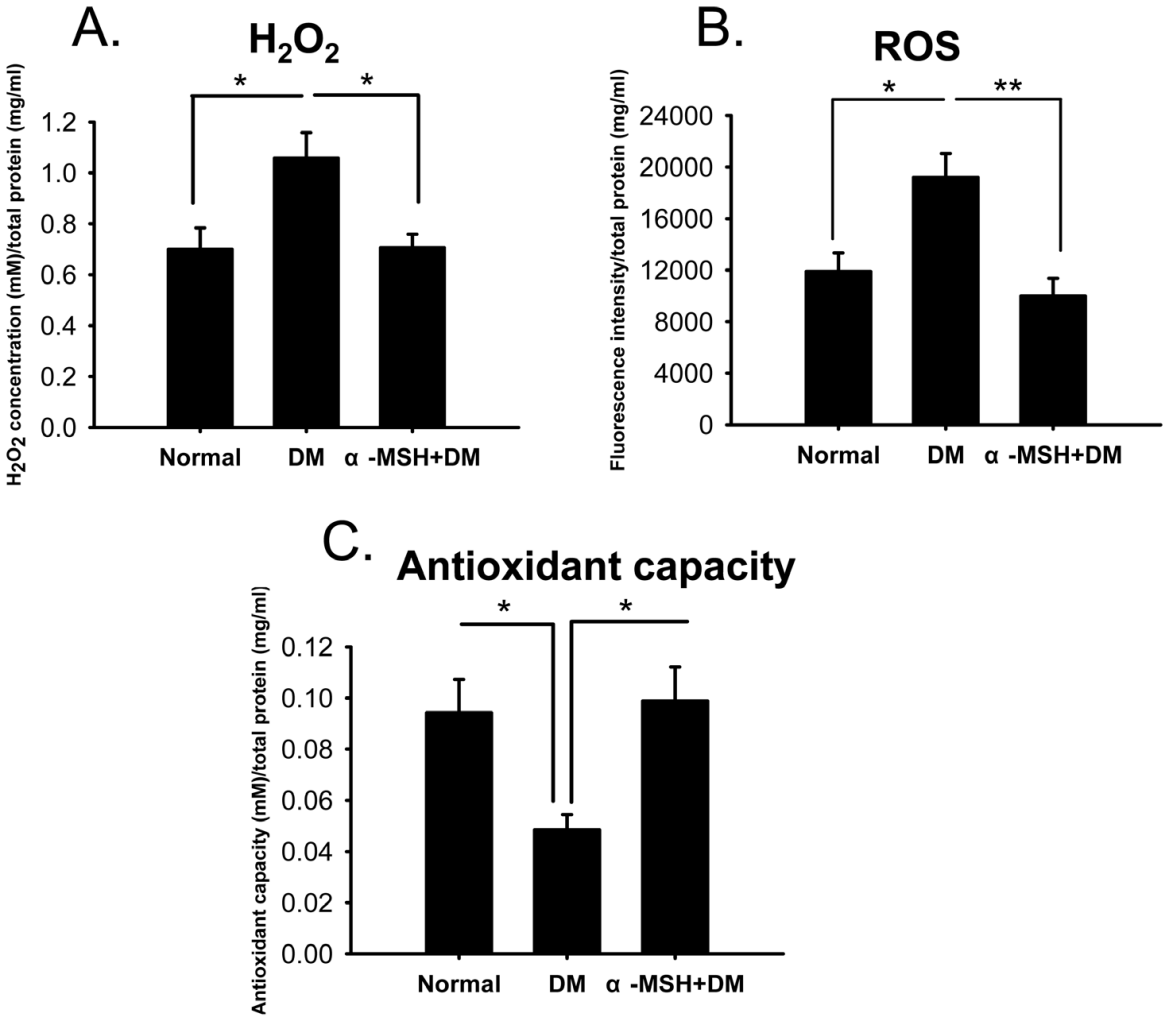
Intravitreal injections of α-MSH normalized the ROS levels and the total anti-oxidative abilities in early diabetic retinas. The levels of H_2_O_2_ (A) and ROS (B), and the total anti-oxidative abilities (C) were measured and normalized to total protein concentrations in the retina samples from experimental groups. n = 5–10/group, * p<0.05, ** p<0.01.

Excessive ROS production is one of the hallmarks of oxidative stress. To determine the effects of α-MSH on oxidative stress in the early diabetic retina, the amounts of H_2_O_2_ and ROS and the total antioxidant capacity were measured in retinal homogenates at week 5 after diabetes induction. After normalizing to total protein concentrations, both H_2_O_2_ and ROS levels in diabetic retinas were elevated 1.5-fold compared with those in normal controls (Fig. 2A and B, both *p*<0.05, DM vs. Normal), whereas the total antioxidant capacity in diabetic retinas was reduced to 56% of the capacity in the normal counterparts (Fig. 2C, *p*<0.05, DM vs. Normal). Intravitreal injections of α-MSH significantly suppressed H_2_O_2_ and ROS levels (Fig. 2A and B, DM vs. α-MSH+DM, *p*<0.05 for H_2_O_2_; *p*<0.01 for ROS), whereas they promoted the total antioxidant capacity (Fig. 2C, DM vs. α-MSH+DM, *p*<0.05) in diabetic retinas. The levels of H_2_O_2_ and ROS and the total antioxidant capacity in α-MSH-treated diabetic retinas were comparable to those in normal ones (Fig. 2A, B, and C, α-MSH+DM vs. normal, *p* = 0.949 for H_2_O_2_, *p* = 0.357 for ROS, *p* = 0.812 for total antioxidant capacity). The results showed that intravitreal injections of α-MSH normalized the ROS levels and the antioxidant capacity in the retinas of early diabetic rats, suggesting the anti-oxidative effects of this peptide.

### α-MSH inhibited apoptosis in trypsin-digested vascular preparation of early diabetic retinas

**Figure 3:**
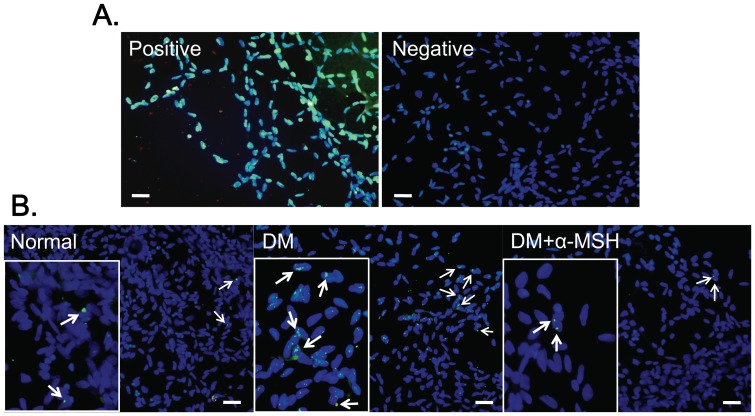
α-MSH administration substantially reduced the number of apoptotic cells in the trypsin-digested diabetic retina preparation. TUNEL staining images were merged with those of DAPI counterstaining. The positive and negative controls for TUNEL staining are shown in (A). The TUNEL staining images of normal control (normal), diabetes (DM), and diabetes with α-MSH intervention (α-MSH+DM) are shown in (B); the insets are the amplified portion of the images for better visualization. n = 8/group, scale bar, 20 μm.

The typical structural changes in retinal microvessels, such as acellular capillary [40], [41] and thickening of capillary basement membrane [40], [42], are rarely detected in STZ-induced diabetic animals by conventional hematoxylin and eosin staining of trypsin-digested preparations of the retinal vascular network until 3 m after diabetes induction. Therefore, the conventional trypsin digestion of rat retinas was followed by TUNEL staining to detect apoptotic cells in retinal vessels at week 6 after diabetic induction in this study. Because the trypsin-digested preparation is the retinal vascular network that contains endothelial cells, pericytes, and smooth muscle cells [40], [43], the cells in the retinal vascular preparation are collectively known as the retinal vascular cells. These cells were counterstained with DAPI to outline the macro-structures of the retinal vessels. As shown in the positive control, the positive TUNEL signals were condensed, punctuated green fluorescence that colocalized with DAPI staining (Fig. 3A). Few TUNEL-positive signals were observed in normal retinal preparations, whereas the positive signals were substantially increased in early diabetic retinal vascular cells (Fig. 3B). Treatment with α-MSH dramatically decreased the TUNEL-positive signals (Fig. 3B) in the retinal vascular cells, suggesting that the α-MSH administration rescued the retinal vascular cells from apoptosis in early DR.

### α-MSH inhibited apoptosis in the neurosensory retinas of diabetic rats

**Figure 4:**
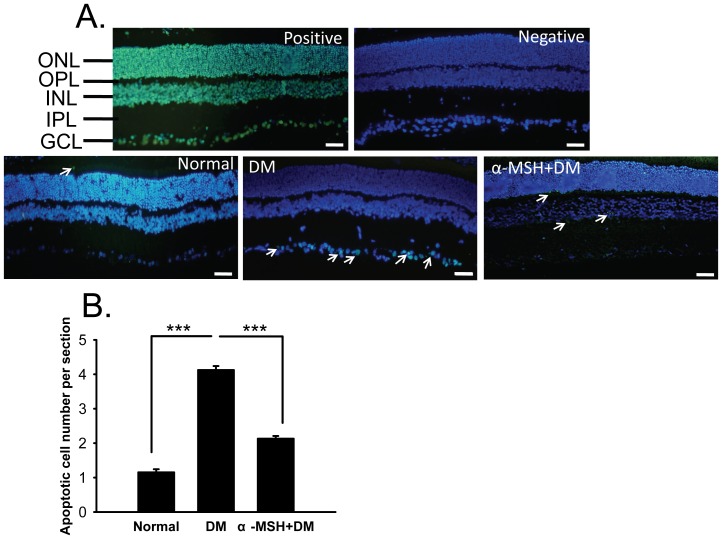
α-MSH significantly reduced the number of apoptotic cells in the neuroretina of diabetic rats. Retinal cryosections were subjected to TUNEL staining. The merged images of TUNEL staining and DAPI counterstaining are shown in (A). The positive and negative controls for TUNEL staining are shown in the upper panel of (A). Representative images for normal, DM and α-MSH+DM groups are shown in the lower panel of (A). The apoptotic cells are indicated by white arrows. The estimated representation of the number of apoptotic cells per section is shown in (B). n = 5–6/group, *** p<0.001. Scale bar, 50 μm.

The apoptotic cells in the neurosensory retina were also detected using TUNEL staining in retinal cryosections. Almost no apoptotic cells were found in normal retinas (Fig. 4A), whereas a significantly higher number of apoptotic cells were detected in the inner retina, particularly in the ganglion cell layer (GCL) of diabetic retinas (Fig. 4A and B, DM vs. normal, *p*<0.001). These observations are consistent with previous reports [44]–[46]. Importantly, intravitreal injections of α-MSH reduced the number of apoptotic cells per section in these retinas to 52% of that in the diabetic retinas without treatment (Fig. 4B, α-MSH+DM vs. DM, *p*<0.001). These results suggest that intravitreal administration of α-MSH reduced the number of apoptotic cells in the early diabetic neuroretina.

### α-MSH ameliorated ultrastructural changes in early diabetic retinas

**Figure 5:**
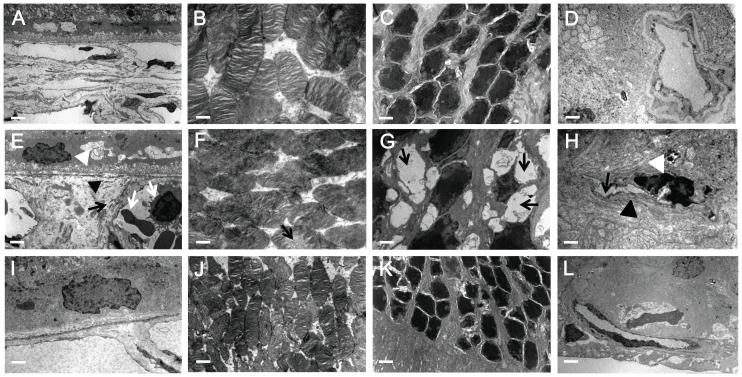
α-MSH ameliorated ultrastructural changes in the early diabetic retina. TEM images of the normal retina (A–D), the diabetic retina (E–H), and the diabetic retina with α-MSH intervention (I–L) are shown. In the diabetic retina (E–H), collagen fibers of the Bruch's membrane were broken and protruded to the capillary lumens (E, black arrow head), basement membranes of the choroid capillaries appeared thickened and laminated (E, black arrow), and the capillary lumen was congested with blood cells (E, white arrows). Bubbles (F, black arrow) and medullary scale bodies (G, black arrows) were observed in the photoreceptor outer segments and outer nuclear layer, respectively. The lumen of the inner retina capillary was severely congested (H, black arrow) by the deformed red blood cells (H, white arrow head), and the basement membrane appeared swelled (H, black arrow head). The ultrastructures of the α-MSH-treated diabetic retinas were greatly improved (J–M) and similar to those in the normal controls (A–D), n = 5/group. Scale bar, 500 nm.

The structural changes in the early diabetic rat retina were subtle; therefore, transmission electron microscopy was employed to detect the protective effects of α-MSH on ultrastructural changes in diabetic retinas. In the week-6 diabetic retinas, Bruch's membrane close to the capillary was swollen. The collagen fibers were dispersed and broken, and they protruded to the capillary lumens (Fig. 5E, black arrow head). The basement membrane of choroid capillaries appeared thickened and laminated (Fig. 5E, black arrow), and the capillary lumen was congested by blood cells (Fig. 5E, white arrows). In the neuroretina, bubbles (Fig. 5F, black arrow) and medullary scale bodies (Fig. 5G, black arrows) were observed in the photoreceptor outer segments and outer nuclear layer, respectively, indicating photoreceptor cell damage. More notably, the lumen of the inner retina capillary was severely congested (Fig. 5H, black arrow) by deformed red blood cells (Fig. 5H, white arrow head), and the basement membrane appeared swelled (Fig. 5H, black arrow head). On the other hand, the ultrastructures of the diabetic retinas treated with α-MSH were substantially improved (Fig. 5I, J, K, L) and exhibited structures similar to those observed in the normal controls (Fig. 5A, B, C, D).

### α-MSH corrected aberrant gene expression changes in diabetic retinas

**Figure 6:**
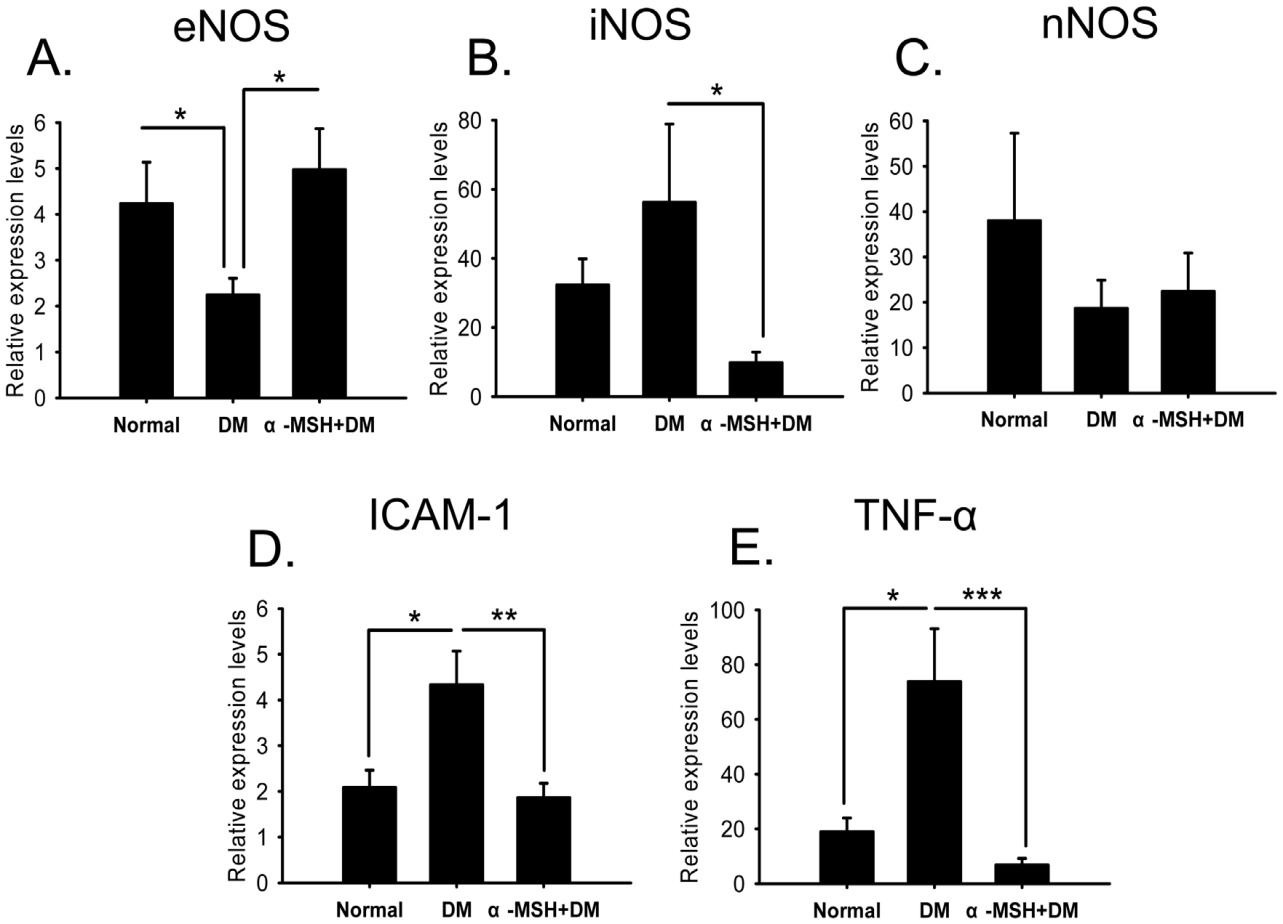
Intravitreal injections of α-MSH corrected aberrant gene expression in the diabetic retina. The relative mRNA expression levels of *eNOS* (A), *iNOS* (B), *nNOS* (C), *ICAM-1* (D), and *TNF-α* (E) in the retinal samples were examined by real-time RT-PCR and normalized to those of *GAPDH*. n = 5–7/group, * p<0.05, ** p<0.01, *** p<0.001.

To understand the protective effects of α-MSH on early diabetic retinas at the molecular level, we performed gene expression analyses in retinas of several factors involved in DR pathogenesis. eNOS is responsible for the constitutive generation of nitric oxide (NO) and has been shown to play a protective role in DR [42]. Consistent with this finding, the *eNOS* mRNA levels in diabetic retinas were reduced 48% compared with those in their normal counterparts (Fig. 6A, *p*<0.05, DM vs. Normal). The intravitreal injections of α-MSH resulted in a restoration of *eNOS* transcript levels (Fig. 6A, *p*<0.05, α-MSH+DM vs. DM). *iNOS* is believed to produce a large amount of NO in response to external stimuli and, hence, to be a pro-oxidative factor. The expression of *iNOS* was up-regulated in diabetic retinas as expected (Fig. 6B), and α-MSH injections substantially suppressed the transcript levels of *iNOS* (Fig. 6B, *p*<0.05, α-MSH+DM vs. DM). The transcript levels of *nNOS* exhibited no significant changes (Fig. 6C). The expression of *ICAM-1* and *TNF-α*, two pro-inflammatory factors, exhibited similar expression patterns. Both mRNA levels were significantly elevated in early diabetic retinas (Fig. 6D and E, both *p*<0.05, DM vs. normal) and were dramatically reduced following α-MSH treatment (Fig. 6D and E, α-MSH+DM vs. DM, *p*<0.01 for *ICAM-1*, *p*<0.05 for *TNF-α*). Therefore, these results suggest that the early diabetic condition induced aberrant gene expression changes (down-regulating the protective factor and up-regulating the pro-oxidative and pro-inflammatory factors), whereas intravitreal injections of α-MSH corrected these aberrations and ameliorated the pro-inflammatory and pro-oxidative molecular environment in the retina of STZ-induced diabetic rats.

### α-MSH inhibited the up-regulation of the transcription factor *Foxo4* in early diabetic retinas

**Figure 7:**
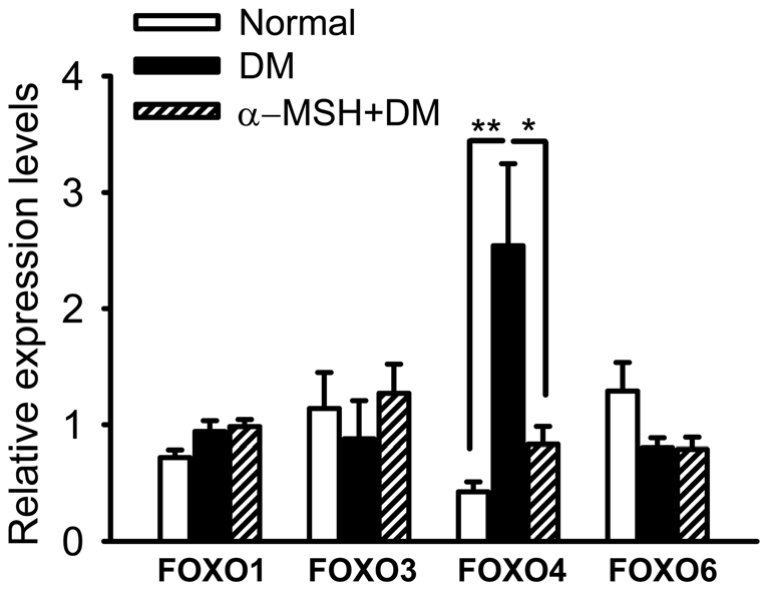
α-MSH inhibited the up-regulation of *Foxo4* in the retina of diabetic rats. The relative expression of *Foxo1*, *Foxo3*, *Foxo4*, and *Foxo6* at the transcript level in the diabetic and normal retinas were examined by real-time RT-PCR and normalized to those of *GAPDH*. The expression of *Foxo4*, but not the other *Foxo* genes, was significantly up-regulated in diabetic retinas compared with the normal controls, and α-MSH treatments inhibited the up-regulation. n = 5–7/group, * p<0.05, ** p<0.01.

To further explore the molecular mechanisms underlying the protective effects of α-MSH, the expression of *Foxo* transcription factor family was examined in the retina of diabetic rats. The real-time PCR results showed that all of the rat *Foxo* genes, *Foxo1*, *3*, *4*, *6*, were detected in the retina; however, only *Foxo4* mRNA levels were up-regulated 6.0-fold in diabetic retinas compared with those in normal retinas (Fig. 7, *p*<0.01, DM vs. normal). More importantly, this up-regulation was dramatically inhibited by intravitreal administration of α-MSH (Fig. 7, *p*<0.05, DM vs. α-MSH+DM), although *Foxo4* mRNA levels in the α-MSH-treated diabetic retinas were moderately higher than the normal ones (Fig. 7, *p* = 0.03, α-MSH+DM vs. normal). The expression of other *Foxo* genes exhibited no significant changes in the diabetic retinas with or without α-MSH intervention (Fig. 7, all *p*>0.05 for *Foxo1*, *3*, and *6*, DM vs. normal, α-MSH+DM vs. normal). These results suggest that the expression of *Foxo4* in the retina is under the regulation of the STZ-induced diabetic condition and α-MSH intervention.

### α-MSH inhibited HG-induced *Foxo4* up-regulation in retinal vascular endothelial cells

**Figure 8:**
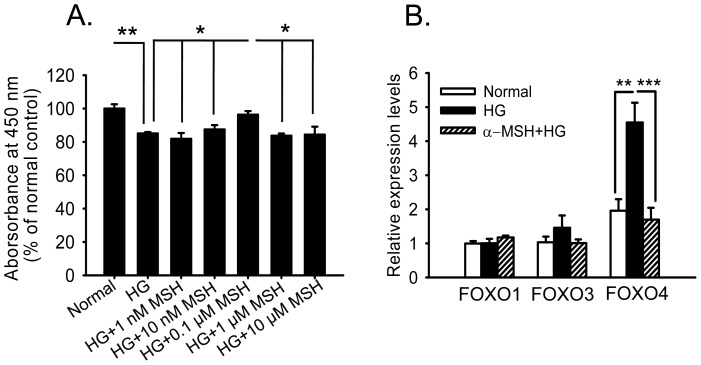
α-MSH inhibited *Foxo4* up-regulation in retinal vascular endothelial cells. The RF/6A cells, a monkey retinal microvessel endothelial cell line, were treated with HG (25 mM) alone or with different concentrations of α-MSH 30 min before and during the HG stimulation. Eight hours following treatment, HG stimulation induced a reduced cell viability that was moderately but significantly boosted by α-MSH at 0.1 μM (A). Then, the cells were exposed to 25 mM HG for 8 h, the mRNA level of *Foxo4*, but not *Foxo1* and *3*, was significantly up-regulated, and this up-regulation was inhibited by incubating the cells with 0.1 μM α-MSH 30 min before and during HG stimulation (B). n = 6/group, * p<0.05, ** p<0.01, *** p<0.001.

To study the direct effects of α-MSH on retinal vascular endothelial cells and further dissect the molecular mechanisms responsible for the protective effects of this peptide, a monkey retinal vascular endothelial cell line RF/6A stimulated with HG was used to recapitulate the STZ-induced hyperglycemic condition in the retinal vessels. The RF/6A cells were stimulated for 8 h with 25 mM HG, the average blood glucose level measured in the STZ-induced diabetic rats (Fig. 1B). Cell viability, as measured with the Cell Counting Kit, was significantly reduced in the HG-simulated endothelial cells. Treatment with α-MSH at 0.1 μM, among all tested concentrations, restored the viability of HG-treated cells to the level similar to that of the normal control cells (Fig. 8A); therefore, this α-MSH concentration was used in the subsequent experiments. In the gene expression analyses, all of the monkey *Foxo* genes except *Foxo6* were expressed in RF/6A cells. Consistent with the results from the diabetic rat retina, *Foxo4* mRNA levels were up-regulated 2.4-fold following HG stimulation compared with that under normal conditions (Fig. 8B, *p*<0.001, HG vs. normal); α-MSH treatment significantly reduced *Foxo4* mRNA levels to levels similar to those under normal conditions (Fig. 8B, *p*<0.001, α-MSH+HG vs. HG; *p* = 0.602, α-MSH+HG vs. normal). The expression levels of *Foxo1* and *3* were not significantly altered after 8 h HG exposure (Fig. 8B, *p* = 0.957 for *Foxo1*, *p* = 0.313 for *Foxo3*, HG vs. normal). These results suggest that α-MSH administration prevented the significant up-regulation of *Foxo4* otherwise observed in the retinal vascular endothelial cells treated with HG only and indicate that the anti-oxidative and anti-apoptotic effects of this peptide observed in the early diabetic retina might be due to its inhibition of *Foxo4* up-regulation.

### *Foxo4* overexpression abrogated the anti-oxidative effects of α-MSH in retinal vascular endothelial cells

**Figure 9:**
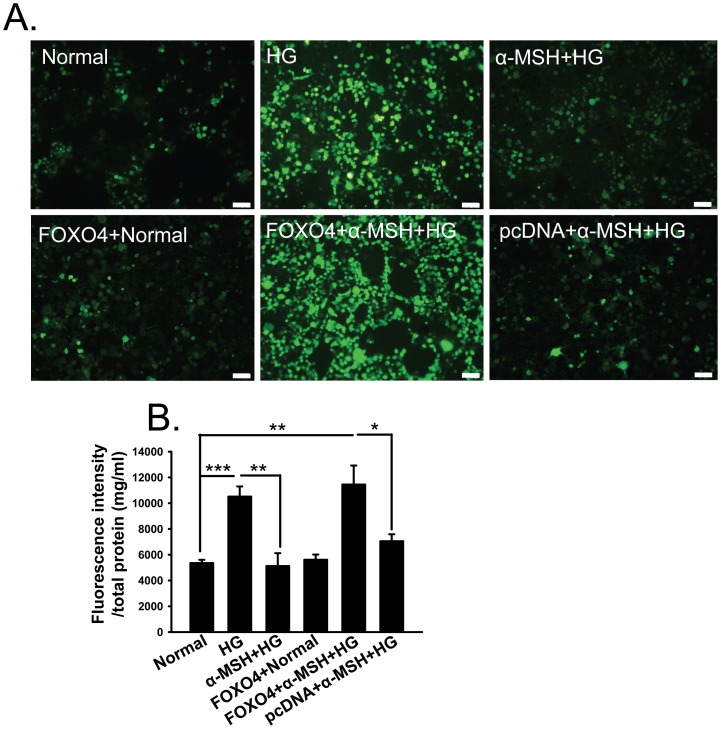
*Foxo4* overexpression abolished anti-oxidative effects of α-MSH in retinal vascular endothelial cells. In the CM-H2DCFDA assay, the fluorescence intensities of cell groups was measured and cell images were captured under a fluorescence microscope. Representative images are shown in (A). The fluorescence intensity was normalized to the total protein concentration of each sample (B). n = 5–10/group, * p<0.05, ** p<0.01, *** p<0.001.

To further corroborate that α-MSH exerted its anti-oxidative effects through preventing *Foxo4* up-regulation, the *Foxo4* cDNA in the HG-stimulated RF/6A cells was cloned into the expression vector pcDNA3.0, and the plasmid expressing *Foxo4* or the empty pcDNA vector was transfected into RF/6A cells. Non-transfected cells were included as controls. After stimulating with HG for 8 h, a cell-permeable oxidative stress indicator, CM-H2DCFDA, was added to the cultures. CM-H2DCFDA is a chemically modified derivative of fluorescein that is non-fluorescent until the acetate groups are removed by intracellular esterases and oxidative reactions [47]. The fluorescence intensity is then normalized to the total protein concentration to correct for potential difference in cell densities. The normalized fluorescence intensity of the cells stimulated with HG was 1.5-fold higher than that of the normal cells (Fig. 9A and B, *p*<0.001, HG vs. normal), which was significantly reduced to normal levels by α-MSH administration (Fig. 9A and B, *p*<0.01, HG vs. α-MSH+HG; *p* = 0.136, α-MSH+HG vs. normal). This result suggests that α-MSH normalizes ROS levels and hence rescues retinal vascular endothelial cells from the oxidative stress induced by HG. In addition, the cells overexpressing *Foxo4* but without HG stimulation exhibited a fluorescence intensity comparable to that in the normal controls (Fig. 9A and B, *p* = 0.595, FOXO4+normal vs. normal), suggesting that *Foxo4* overexpression under normal conditions is not sufficient to render the cells under oxidative stress. More interestingly, HG stimulation in the cells overexpressing *Foxo4* caused a significantly elevated fluorescent intensity despite the presence of α-MSH (Fig. 9A and B, *p*<0.001, FOXO4+α-MSH+HG vs. α-MSH+HG), whereas such a phenomenon was not observed in the cells transfected with the empty pcDNA vector (Fig. 9A and B, *p*<0.01, FOXO4+α-MSH+HG vs. pcDNA+α-MSH+HG; *p* = 0.07, pcDNA+α-MSH+HG vs. α-MSH+HG). These results suggest an interaction between *Foxo4* overexpression and HG stimulation in our culture system, and further indicate that prevention of HG-induced *Foxo4* up-regulation is essential for α-MSH to generate anti-oxidative effects in retinal vascular endothelial cells.

### *Foxo4* overexpression abolished the anti-apoptotic effects of α-MSH in retinal vascular endothelial cells

**Figure 10:**
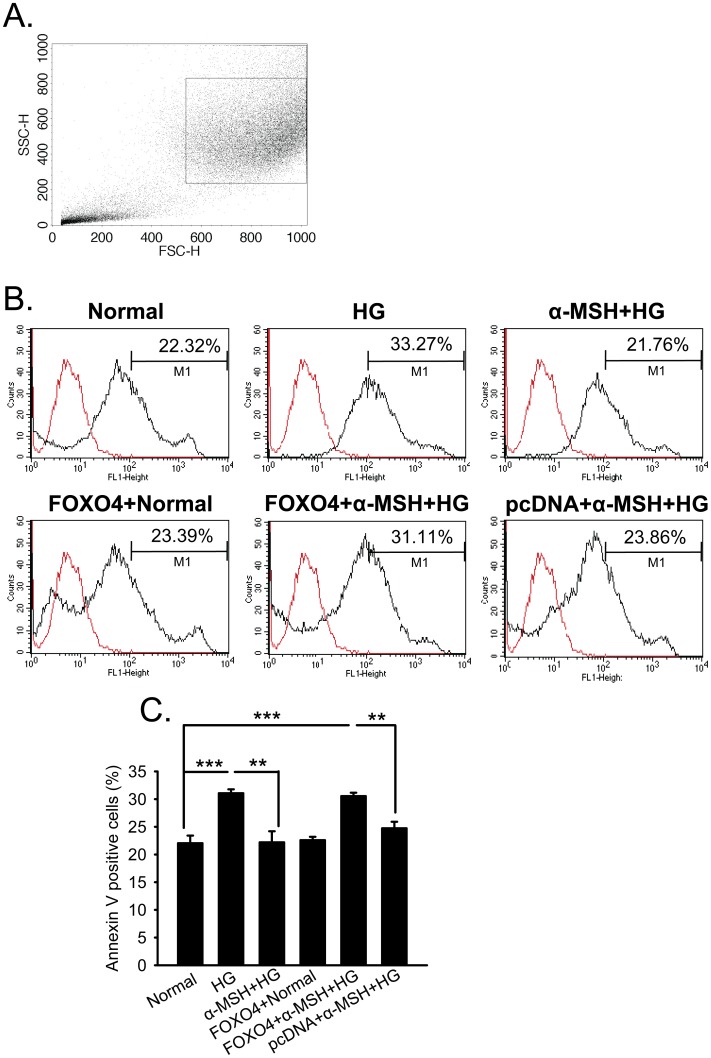
*Foxo4* overexpression abrogated anti-apoptotic effects of α-MSH in retinal vascular endothelial cells. Following various treatments, the cell groups were stained with Alexa 488-annexin V and analyzed by flow cytometry. The dot plot (A) shows the gate selection based on the forward scatter (abscissa) and side scatter (ordinate) of the RF/6A cells, so that the dead cells and cell debris were excluded. The histograms (B) are representative images of the flow cytometry analyses. The abscissa and ordinate represent the fluorescence intensity of Alexa 488-annexin V and cell counts, respectively. The distribution of the non-stained RF/6A cells cultured under the complete culture media was included as a negative control (red line) and used to set the marker M1. The distributions of the treated and stained cells are also shown (black line). The cell populations inside M1 were considered positive for Alexa 488-annexin V staining. The number above M1 indicates the percentage of the cells positive for the Alexa 488-annexin V staining in each representative picture. The Alexa 488-annexin V staining positivity in each treatment group was compared in (C). n = 6/group, ** p<0.01, *** p<0.001.

To confirm the role of FoxO4 in the anti-apoptotic effects of α-MSH in the retinal vascular endothelial cells, the cells overexpressing *Foxo4* or vector control were stained with Alexa 488-annexin V and analyzed by flow cytometry. The percentage of the cells positive for Alexa 488-annexin V was 22.05±3.35% in the cells cultured in serum-free RPMI 1640 media for 8 h; this percentage significantly increased to 31.06±1.72% after incubating in serum-free culture media containing 25 mM glucose (*p*<0.001, HG vs. normal; Fig. 10C). The presence of α-MSH normalized the frequency of Alexa 488-annexin-V-positive cells (*p*<0.001, α-MSH+HG vs. HG; p = 0.96, α-MSH+HG vs. normal; Fig. 10C), suggesting the anti-apoptotic effects of this peptide in retinal vascular endothelial cells stimulated with HG. Similar to the results of the oxidative stress assay, overexpression of *Foxo4* under normal conditions did not significantly alter the Alexa 488-annexin V positivity (Fig. 10C, *p* = 0.726, FOXO4+normal vs. normal); however, under HG stimulation, the anti-apoptotic effect of α-MSH was abolished by overexpressing *Foxo4*, but not by transfecting the empty vector (Fig. 10C, *p*<0.001, FOXO4+α-MSH+HG vs. normal; *p*<0.01, FOXO4+α-MSH+HG vs. α-MSH+HG). In addition to the fact that *Foxo4* overexpression and HG stimulation may interact on apoptosis induction in the retinal vascular endothelial cells, these results also indicate that preventing *FOXO4* up-regulation is necessary for the anti-apoptotic effects of α-MSH in these cells cultured under HG conditions.

## Discussion

In this study, we for the first time showed the anti-oxidative and anti-apoptotic effects of intravitreally administered α-MSH, a naturally occurring ocular peptide, in early DR. Specifically, α-MSH normalized ROS production and antioxidant capacity in the retina (Fig. 2), inhibited apoptosis in the retinal vascular network (Fig. 3) and the neurosensory retina (Fig. 4) in STZ-induced diabetic rats. The ultrastructural damages in choroid and retinal vessels, photoreceptor outer segments, and outer nuclear layer were substantially attenuated (Fig. 5). At the molecular level, α-MSH rectified the aberrant changes in the expression of pro-oxidative and pro-inflammatory factors in the diabetic retina (Fig. 6). These results demonstrate the protective effects of α-MSH in the retina of STZ-induced diabetic rats and suggest the possibility that intravitreal application of α-MSH could be developed into a novel and effective intervention for early DR. With regard to the mechanism underlying these protective effects, gene expression analyses showed that *Foxo4* is the only member of *Foxo* transcription factor family that was significantly up-regulated in diabetic retinas (Fig. 7) and in HG-stimulated retinal vascular endothelial cells (Fig. 8B), and the up-regulation was suppressed by α-MSH (Fig. 7 and 8B). These results indicate that inhibition of the HG-induced *Foxo4* up-regulation in both the retina and culture by α-MSH might be responsible for the protective effects of this peptide. Furthermore, *Foxo4* overexpression by transient transfection abolished the anti-oxidative and anti-apoptotic effects of α-MSH in the retinal vascular endothelial cells exposed to HG (Fig. 9 and 10). The results of these *in vitro* studies suggest that inhibition of HG-induced *Foxo4* up-regulation is essential for the protective effects of α-MSH, and this novel regulatory mechanism of *Foxo4* at the transcriptional level also indicates a new therapeutic target for early DR.

Oxidative stress has been shown to be involved in multiple chronic diseases, including age-related macular degeneration [48], DR [12], Alzheimer's and Parkinson's disease [49]; the therapeutic regimen and mechanistic study against oxidative stress in one disease may provide clues for another. The anti-oxidative effects of α-MSH have been studied in skin diseases. For instance, α-MSH counteracts the UVB irradiation-induced oxidative stress by up-regulating the expression of the transcription factor Nrf2 and its dependent genes through MC1R-mediated activation of cAMP/PKA pathway in skin keratinocytes and melanocytes [25]. In UVA-irradiated melanocytes, α-MSH reduces ROS levels and protects cells from DNA damage through the cAMP/PKA and PI3K/Akt pathway-mediated P53 phosphorylation [24]. Because skin tissue and retina are both developed from the embryonic ectoderm, and *MCRs* are expressed in the diabetic retina (Fig. 1 C and D), we hypothesize that α-MSH may exert anti-oxidative effects in the early diabetic retina. Indeed, our results in STZ-induced diabetic rats showed that retinal levels of H_2_O_2_ and ROS were significantly suppressed, whereas total antioxidant capacity in the retina was promoted by intravitreal injections of this peptide (Fig. 2). In addition, data from HG-stimulated cell cultures confirmed the anti-oxidative effects of α-MSH using a well-recognized oxidative stress indicator CM-H2DCFDA (Fig. 9). Therefore, the local application of α-MSH antagonizes oxidative stress in early DR.

The anti-apoptotic effects of α-MSH may partially result from its anti-oxidative effects in the early diabetic retina. However, it has been shown that α-MSH or its analog can also rescue neurons from apoptosis induced by other insults, including traumatic brain injury caused by controlled cerebral impact [50], cerebral ischemia generated by common carotid artery occlusion [51], and hippocampal excitotoxicity induced by excitatory neurotransmitter glutamate [52]. Indeed, the results from another study in progress in our laboratory showed that α-MSH dose and time-dependently reduced the number of apoptotic cells induced by glutamate both in the retinal explants [53] and in retinas *in vivo* (unpublished data). The results of these studies suggest a wide spectrum of anti-apoptotic function for this peptide. Therefore, we examined the anti-apoptotic effects of α-MSH by TUNEL staining in the retinal vascular preparation (Fig. 3) and neuroretina (Fig. 4) from diabetic rats. TUNEL identifies DNA strand breaks in apoptotic cells. However, cleavage of genomic DNA is a late apoptotic event [54]. We then employed annexin V staining to detect early apoptotic cells in the subsequent cell culture study. Annexin V has a high affinity for phosphatidyl serine that has translocated from the inner to the outer leaflet of the plasma membrane during early apoptosis [55]. The results of annexin V (Fig. 10) and TUNEL (Fig. 3 and 4) staining suggest the protective effects of α-MSH in both early and late apoptosis, respectively. Moreover, examining the expression and activity of the caspases may unravel the α-MSH's anti-apoptotic effects at the molecular level.

With regard to the mechanism underlying the anti-oxidative and anti-apoptotic effects of α-MSH in diabetic retinas, we focused on FoxO4, a transcription factor that plays pivotal roles in oxidative stress [28] and apoptosis [30], [56]. In the retinas of STZ-induced diabetic rats and the retinal vascular endothelial cells exposed to HG (25 mM) for 8 h, we found that *Foxo4* was the only *Foxo* family member that exhibited significant up-regulation, and this up-regulation was inhibited by α-MSH treatment (Fig. 7 and 8B). Therefore, we developed our hypothesis that α-MSH exerts anti-oxidative and anti-apoptotic effects in retinal vascular endothelial cells through inhibiting HG-induced *Foxo4* up-regulation. The results of subsequent experiments supported our hypothesis by showing that *Foxo4* overexpression by transient transfection abrogated the anti-oxidative and anti-apoptotic effects of α-MSH. The results also indicate FoxO4 as a novel target for early DR intervention. However, in a previous study using primary rat retinal vascular endothelial cells that had been treated with HG (25 mM) for 5 d, *Foxo1* mRNA levels were up-regulated [57]. Because the expression of *Foxo* genes in diabetic rat retinas (Fig. 7) exhibited a pattern similar to that in monkey retinal endothelial cells (Fig. 8B), the species difference does not appear to have a major impact on *Foxo* regulation. Indeed, Urbich and colleagues showed that FoxO4 is also the main subtype of FoxO transcription factors in human endothelial progenitor cells, and its inactivation by posttranslational modification suppresses oxidative stress-induced apoptosis [32]. On the other hand, the expression and activity levels of FoxOs are tightly regulated in a temporal-spatial manner [36]. We analyzed gene expression in early diabetic retinas, as 5 weeks after diabetes is a considered short term for diabetic animals. We also studied gene expression in the retinal vascular endothelial cells treated with HG for 8 h, a relatively short time compared with 5 d as in the previous study. Thus, it is reasonable to speculate that *Foxo1* up-regulation might play a major role during long-term HG treatment in the primary retinal vascular endothelial cells, whereas *Foxo4* up-regulation at the transcriptional level might respond to acute HG stimulation both in RF/6A cells and in rat retinas. The mechanistic study sheds light on a novel *Foxo4* regulatory mechanism underpinning the protective effects of α-MSH.

The ultimate goal of this study is to develop an effective intervention approach to early DR, the route of drug delivery is then a primary facet to consider. DR is a localized disease of the eye; therefore, intravitreal injection of α-MSH has the following advantages over systemic delivery. First, intravitreal administration may reduce drug dosage. α-MSH has a short half-life in rat [58] and human [59] plasma; large doses and multiple applications of this peptide are needed to overcome rapid clearance and untargeted organ catabolism via systemic delivery. However, with the injection of α-MSH into the vitreous cavity of diabetic rats, a small and confined space, the peptide gains direct access to retina, the main DR lesion site. Therefore, the dose and frequency of α-MSH administration may be reduced. Moreover, the acetylated form of α-MSH used in our study may have a prolonged half-life compared with the unmodified counterpart [60]. Therefore, we performed two intravitreal injections of α-MSH at 10 μg to the diabetic rats; comparable doses and frequencies have been employed in a rodent model of uveitis for subconjunctival injection [61]. Second, MCRs are distributed throughout the body, with each subtype mediating different intracellular signaling pathways and biological functions [14]. α-MSH, delivered by either intravenous or intraperitoneal injection, may incur side effects by binding to MCRs at different organs. Indeed, studies have shown that acute and chronic activation of MC3/4R at peripheral organs, such as heart, blood vessels, and kidney, cause an elevation of blood pressure and heart rate [14], [62]–[64]. Therefore, because it entails delivering a reduced dose into a restricted space, the intravitreal injection of α-MSH may reduce side effects. In fact, we observed no changes in physiological parameters, including body weight and blood glucose levels, in the diabetic rats that had been intravitreally injected twice with α-MSH (Fig. 1A and B), suggesting that the systemic effects of the injected α-MSH are minimal.

Because all major *MCRs* are expressed in the diabetic retina (Fig. 1C and D), and α-MSH has pleiotropic affinity to all the MCRs, it would be interesting in future studies to ascertain which receptor subtype mainly, if not solely, mediates the protective effects of α-MSH in the early diabetic retina. Then, this peptide could be chemically modified with augmented durability and binding specificity to that receptor subtype, aiming to increasing therapeutic efficacy and reducing the potential side effects of this peptide.

In summary, intravitreal injections of α-MSH demonstrated anti-oxidative and anti-apoptotic effects in the retina of STZ-induced diabetic rats, supporting the possibility that α-MSH or its chemical derivatives can be developed into an effective and novel intervention approach for early DR. The mechanism underlying the protective effects of this peptide in retinal vascular endothelial cells reveals a novel regulatory mechanism involving FOXO4 at the transcriptional level.
